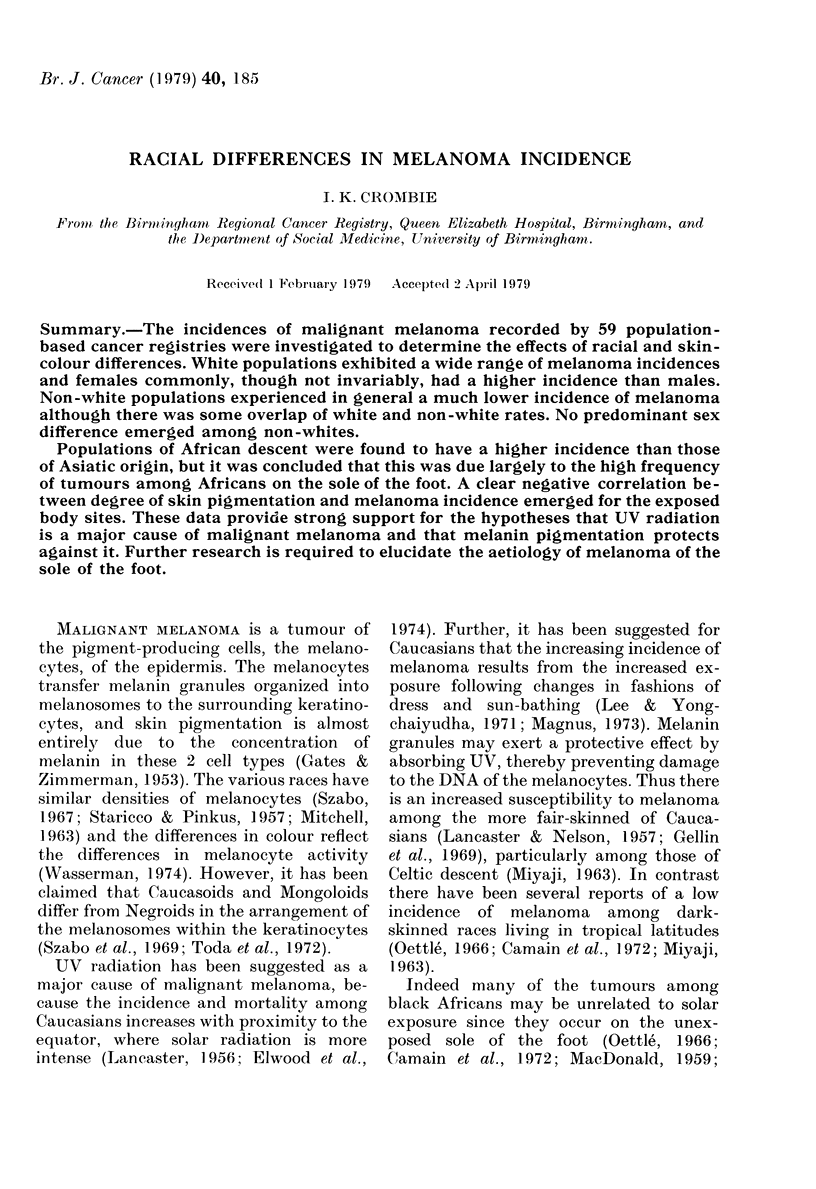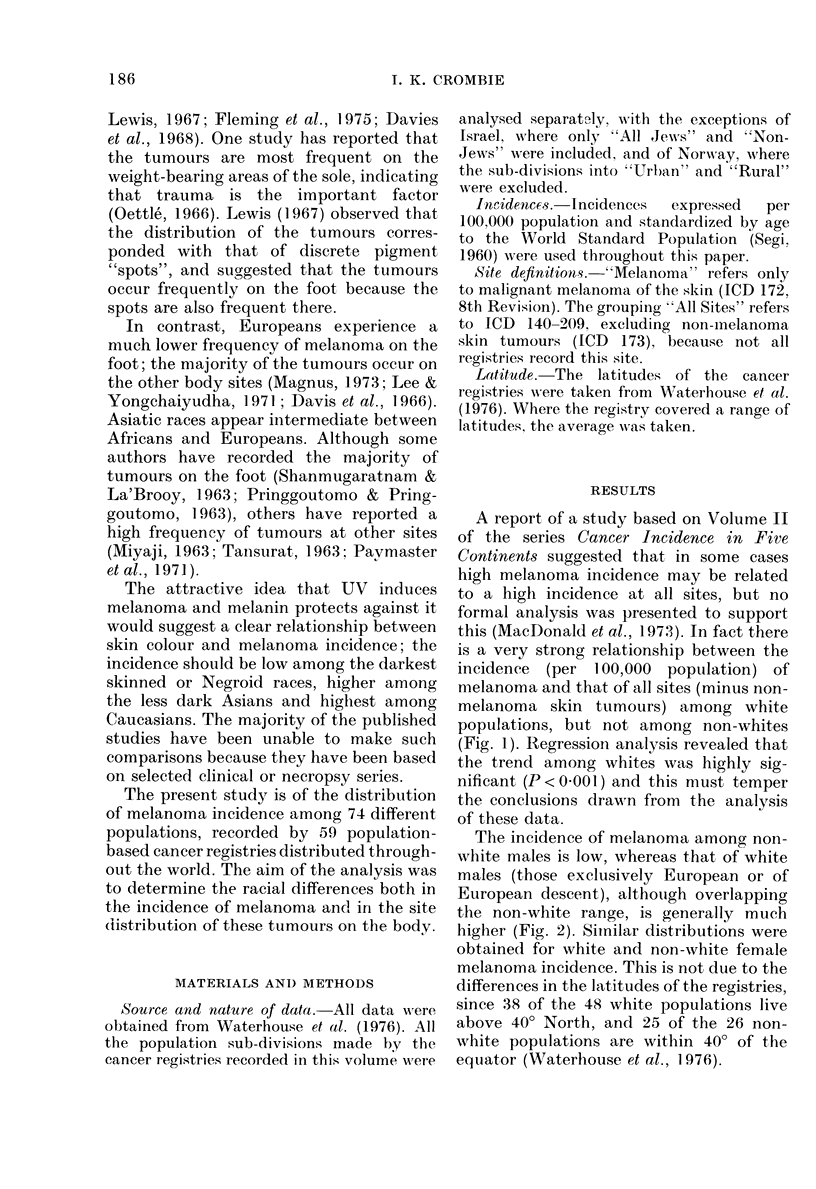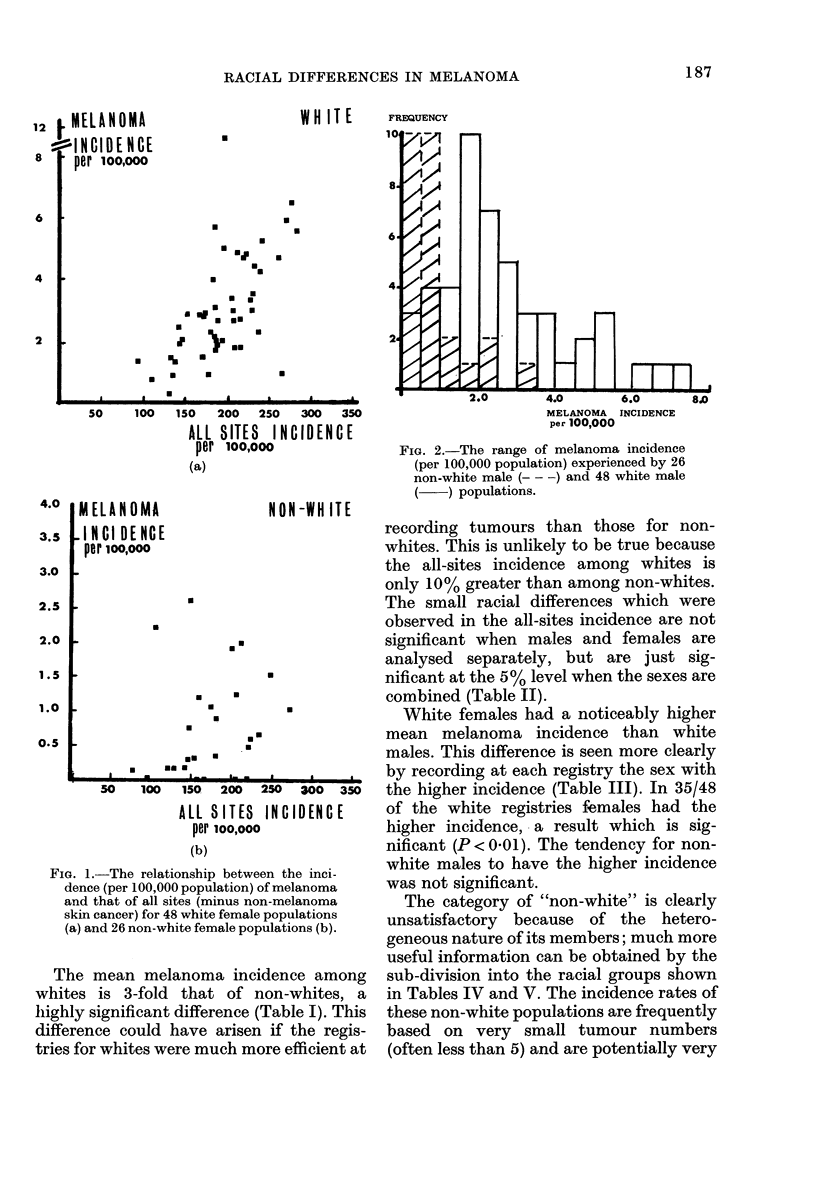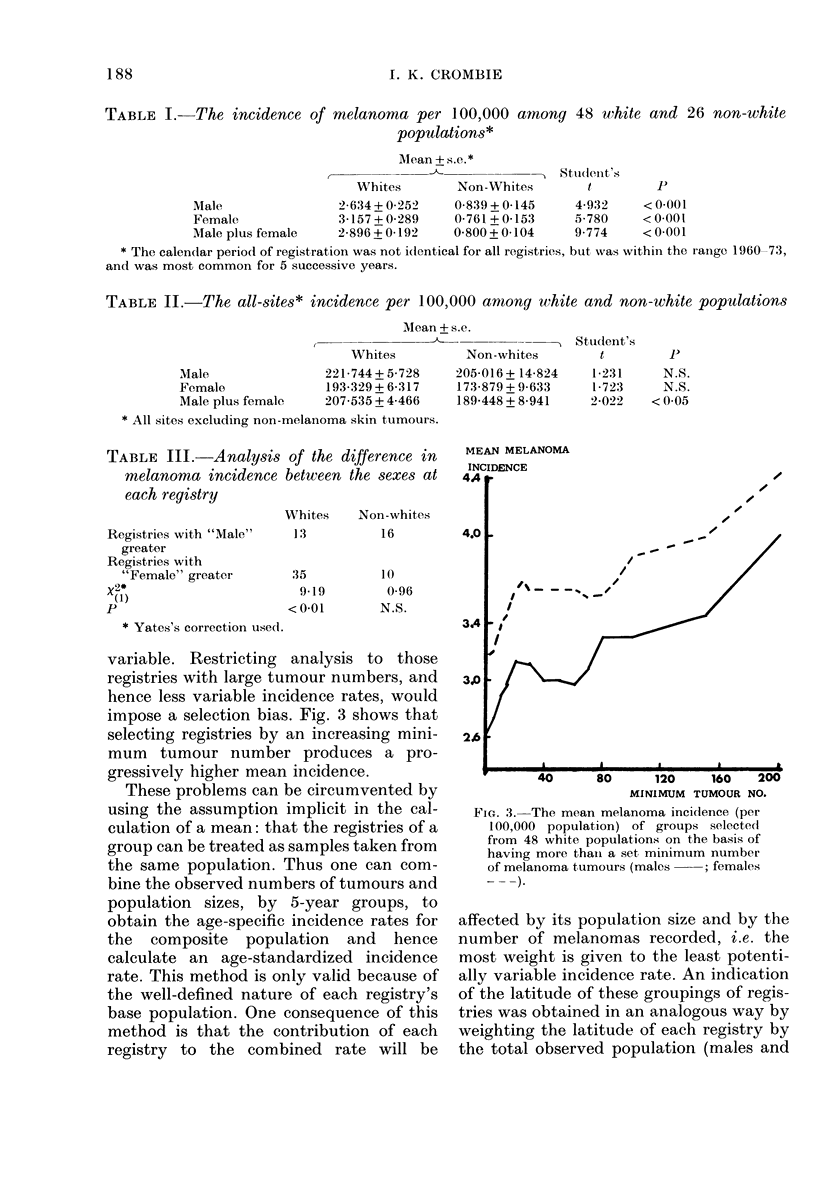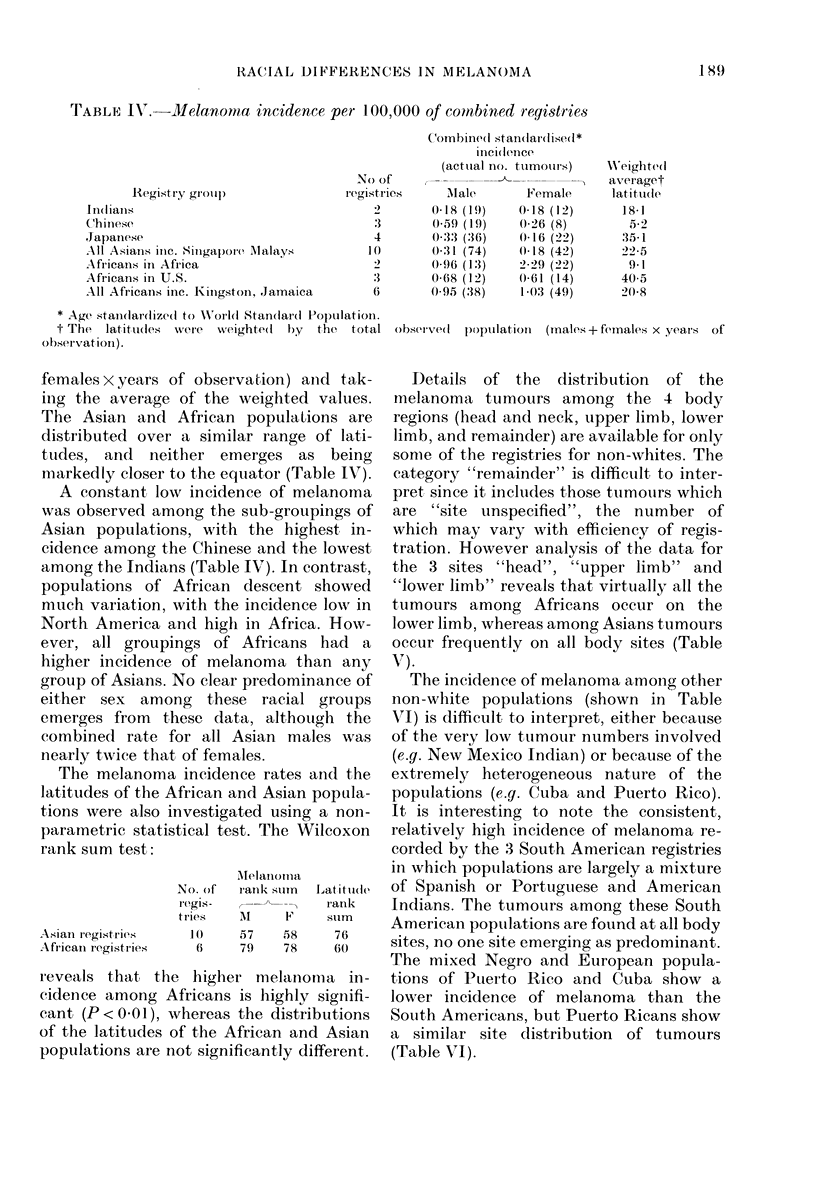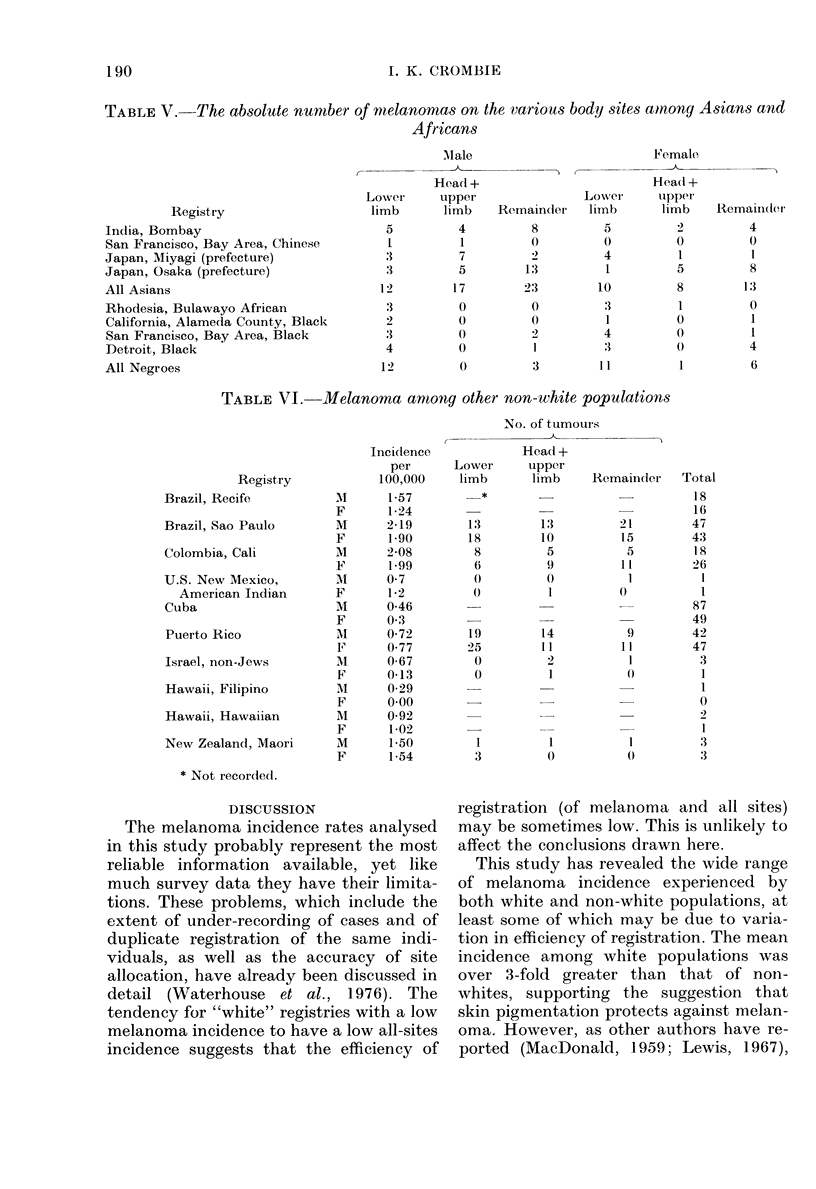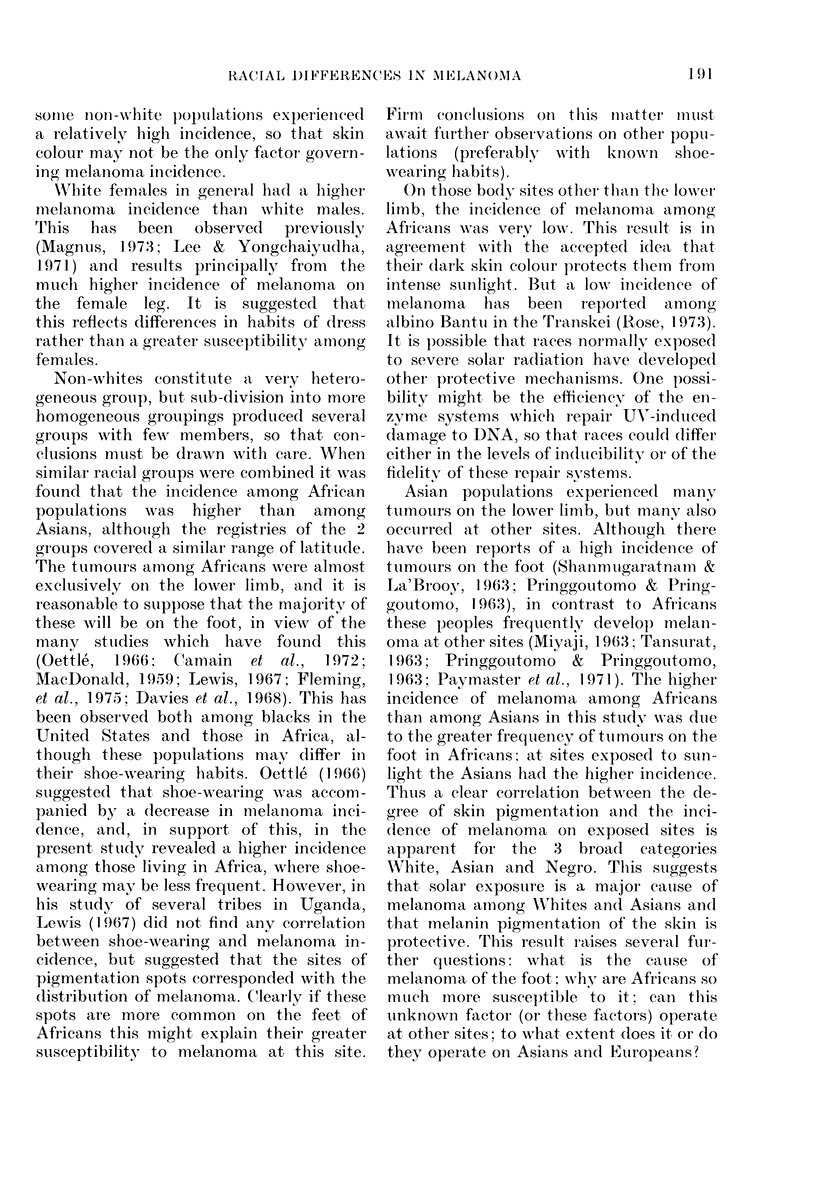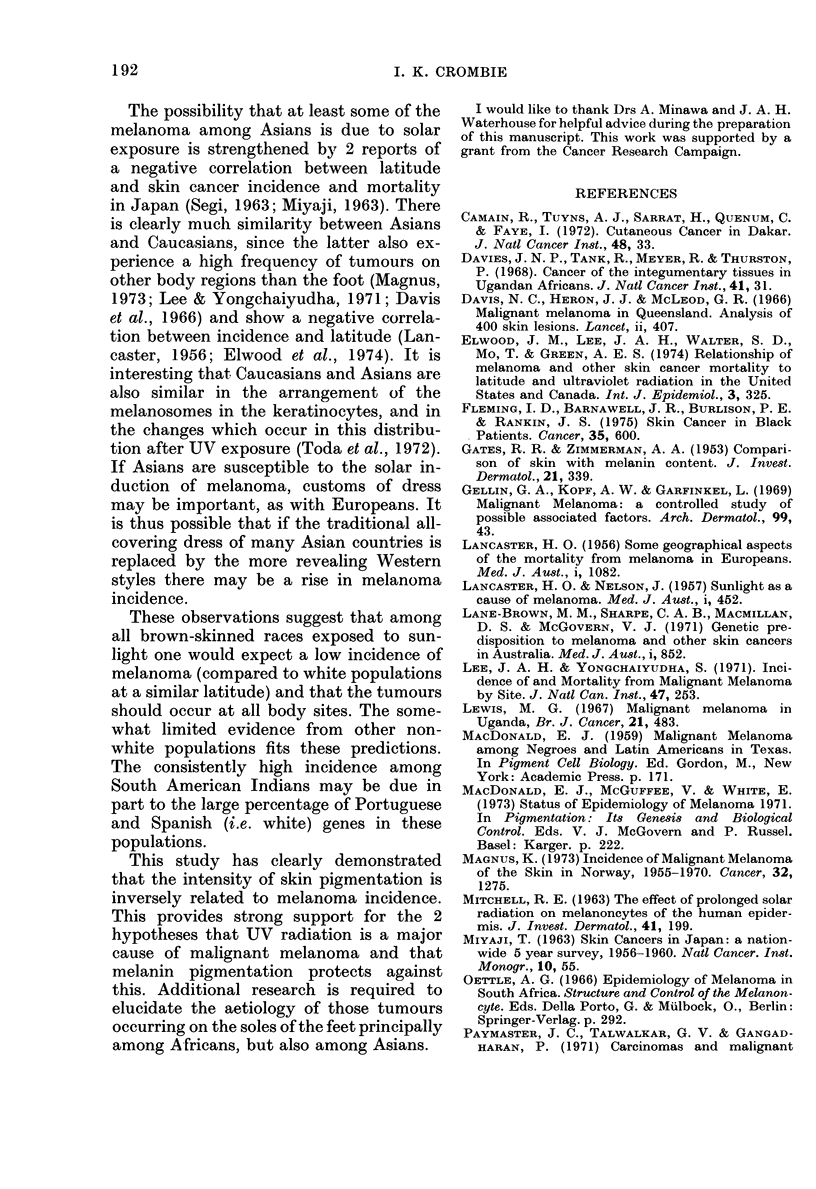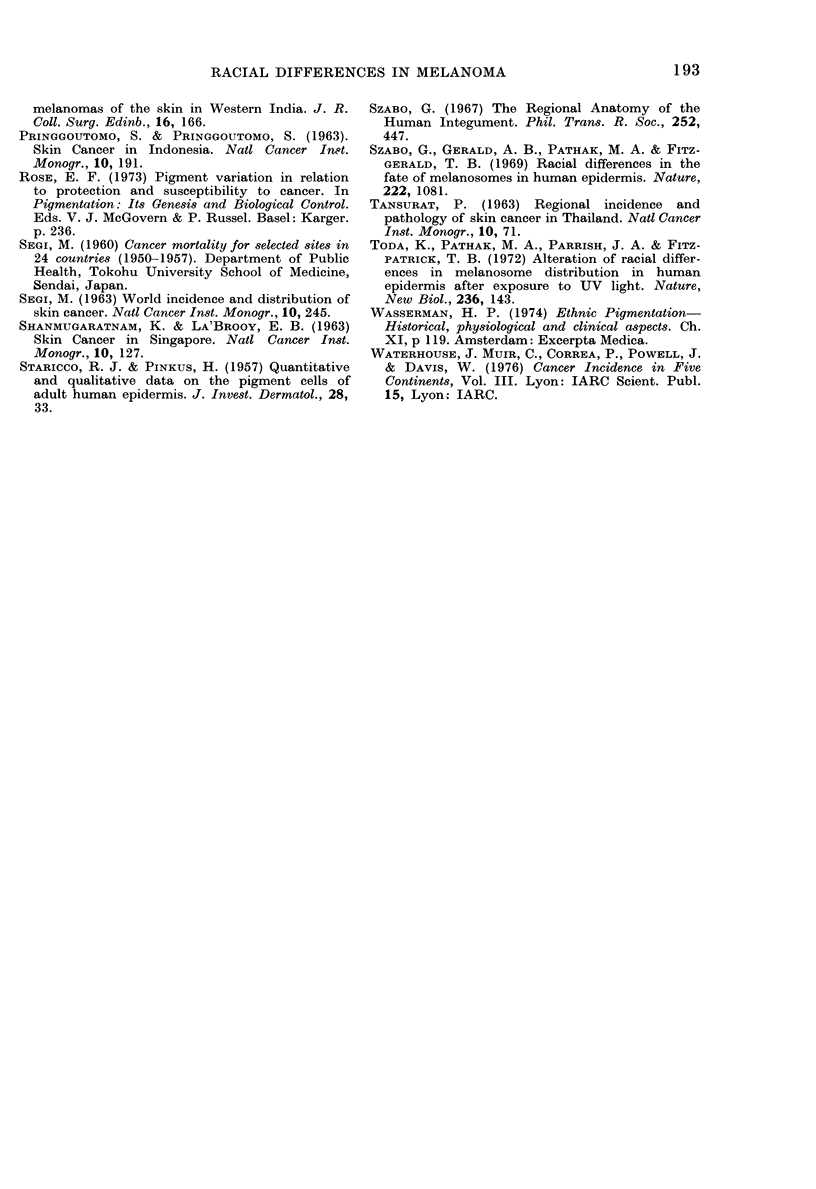# Racial differences in melanoma incidence.

**DOI:** 10.1038/bjc.1979.165

**Published:** 1979-08

**Authors:** I. K. Crombie

## Abstract

The incidences of malignant melanoma recorded by 59 population-based cancer registries were investigated to determine the effects of racial and skin-colour differences. White populations exhibited a wide range of melanoma incidences and females commonly, though not invariably, had a higher incidence than males. Non-white populations experienced in general a much lower incidence of melanoma although there was some overlap of white and non-white rates. No predominant sex difference emerged among non-whites. Populations of African descent were found to have a higher incidence than those of Asiatic origin, but it was concluded that this was due largely to the high frequency of tumours among Africans on the sole of the foot. A clear negative correlation between degree of skin pigmentation and melanoma incidence emerged for the exposed body sites. These data provide strong support for the hypotheses that UV radiation is a major cause of malignant melanoma and that melanin pigmentation protects against it. Further research is required to elucidate the aetiology of melanoma of the sole of the foot.


*Br. J. Cancer* (1979) **40**, 185

### RACIAL DIFFERENCES IN MELANOMA INCIDENCE

I. K. CROMBIE

*From the Birmingham Regional Cancer Registry, Queen Elizabeth Hospital, Birmingham, and the Department of Social Medicine, University of Birmingham.*

Received 1 February 1979 Accepted 2 April 1979

**Summary.—The incidences of malignant melanoma recorded by 59 population-based cancer registries were investigated to determine the effects of racial and skin-colour differences. White populations exhibited a wide range of melanoma incidences and females commonly, though not invariably, had a higher incidence than males. Non-white populations experienced in general a much lower incidence of melanoma although there was some overlap of white and non-white rates. No predominant sex difference emerged among non-whites.**

**Populations of African descent were found to have a higher incidence than those of Asiatic origin, but it was concluded that this was due largely to the high frequency of tumours among Africans on the sole of the foot. A clear negative correlation between degree of skin pigmentation and melanoma incidence emerged for the exposed body sites. These data provide strong support for the hypotheses that UV radiation is a major cause of malignant melanoma and that melanin pigmentation protects against it. Further research is required to elucidate the aetiology of melanoma of the sole of the foot.**

Malignant melanoma is a tumour of the pigment-producing cells, the melanocytes, of the epidermis. The melanocytes transfer melanin granules organized into melanosomes to the surrounding keratinocytes, and skin pigmentation is almost entirely due to the concentration of melanin in these 2 cell types (Gates & Zimmerman, 1953). The various races have similar densities of melanocytes (Szabo, 1967; Staricco & Pinkus, 1957; Mitchell, 1963) and the differences in colour reflect the differences in melanocyte activity (Wasserman, 1974). However, it has been claimed that Caucasoids and Mongoloids differ from Negroids in the arrangement of the melanosomes within the keratinocytes (Szabo *et al.*, 1969; Toda *et al.*, 1972).

UV radiation has been suggested as a major cause of malignant melanoma, because the incidence and mortality among Caucasians increases with proximity to the equator, where solar radiation is more intense (Lancaster, 1956; Elwood *et al.*, 1974). Further, it has been suggested for Caucasians that the increasing incidence of melanoma results from the increased exposure following changes in fashions of dress and sun-bathing (Lee & Yongchaiyudha, 1971; Magnus, 1973). Melanin granules may exert a protective effect by absorbing UV, thereby preventing damage to the DNA of the melanocytes. Thus there is an increased susceptibility to melanoma among the more fair-skinned of Caucasians (Lancaster & Nelson, 1957; Gellin *et al.*, 1969), particularly among those of Celtic descent (Miyaji, 1963). In contrast there have been several reports of a low incidence of melanoma among dark-skinned races living in tropical latitudes (Oettlé, 1966; Camain *et al.*, 1972; Miyaji, 1963).

Indeed many of the tumours among black Africans may be unrelated to solar exposure since they occur on the unexposed sole of the foot (Oettlé, 1966; Camain *et al.*, 1972; MacDonald, 1959;

Lewis, 1967; Fleming *et al.*, 1975; Davies *et al.*, 1968). One study has reported that the tumours are most frequent on the weight-bearing areas of the sole, indicating that trauma is the important factor (Oettlé, 1966). Lewis (1967) observed that the distribution of the tumours corresponded with that of discrete pigment "spots", and suggested that the tumours occur frequently on the foot because the spots are also frequent there.

In contrast, Europeans experience a much lower frequency of melanoma on the foot; the majority of the tumours occur on the other body sites (Magnus, 1973; Lee & Yongchaiyudha, 1971; Davis *et al.*, 1966). Asiatic races appear intermediate between Africans and Europeans. Although some authors have recorded the majority of tumours on the foot (Shanmugaratnam & La'Brooy, 1963; Pringgoutomo & Pringgoutomo, 1963), others have reported a high frequency of tumours at other sites (Miyaji, 1963; Tansurat, 1963; Paymaster *et al.*, 1971).

The attractive idea that UV induces melanoma and melanin protects against it would suggest a clear relationship between skin colour and melanoma incidence; the incidence should be low among the darkest skinned or Negroid races, higher among the less dark Asians and highest among Caucasians. The majority of the published studies have been unable to make such comparisons because they have been based on selected clinical or necropsy series.

The present study is of the distribution of melanoma incidence among 74 different populations, recorded by 59 population-based cancer registries distributed throughout the world. The aim of the analysis was to determine the racial differences both in the incidence of melanoma and in the site distribution of these tumours on the body.

### MATERIALS AND METHODS

*Source and nature of data.*—All data were obtained from Waterhouse *et al.* (1976). All the population sub-divisions made by the cancer registries recorded in this volume were analysed separately, with the exceptions of Israel, where only "All Jews" and "Non-Jews" were included, and of Norway, where the sub-divisions into "Urban" and "Rural" were excluded.

*Incidences.*—Incidences expressed per 100,000 population and standardized by age to the World Standard Population (Segi, 1960) were used throughout this paper.

*Site definitions.*—"Melanoma" refers only to malignant melanoma of the skin (ICD 172, 8th Revision). The grouping "All Sites" refers to ICD 140–209, excluding non-melanoma skin tumours (ICD 173), because not all registries record this site.

*Latitude.*—The latitudes of the cancer registries were taken from Waterhouse *et al.* (1976). Where the registry covered a range of latitudes, the average was taken.

### RESULTS

A report of a study based on Volume II of the series *Cancer Incidence in Five Continents* suggested that in some cases high melanoma incidence may be related to a high incidence at all sites, but no formal analysis was presented to support this (MacDonald *et al.*, 1973). In fact there is a very strong relationship between the incidence (per 100,000 population) of melanoma and that of all sites (minus non-melanoma skin tumours) among white populations, but not among non-whites (Fig. 1). Regression analysis revealed that the trend among whites was highly significant ($P < 0{\cdot}001$) and this must temper the conclusions drawn from the analysis of these data.

The incidence of melanoma among non-white males is low, whereas that of white males (those exclusively European or of European descent), although overlapping the non-white range, is generally much higher (Fig. 2). Similar distributions were obtained for white and non-white female melanoma incidence. This is not due to the differences in the latitudes of the registries, since 38 of the 48 white populations live above 40° North, and 25 of the 26 non-white populations are within 40° of the equator (Waterhouse *et al.*, 1976).

FIG. 1.—The relationship between the incidence (per 100,000 population) of melanoma and that of all sites (minus non-melanoma skin cancer) for 48 white female populations (a) and 26 non-white female populations (b).

FIG. 2.—The range of melanoma incidence (per 100,000 population) experienced by 26 non-white male (- - -) and 48 white male (——) populations.

The mean melanoma incidence among whites is 3-fold that of non-whites, a highly significant difference (Table I). This difference could have arisen if the registries for whites were much more efficient at recording tumours than those for non-whites. This is unlikely to be true because the all-sites incidence among whites is only 10% greater than among non-whites. The small racial differences which were observed in the all-sites incidence are not significant when males and females are analysed separately, but are just significant at the 5% level when the sexes are combined (Table II).

White females had a noticeably higher mean melanoma incidence than white males. This difference is seen more clearly by recording at each registry the sex with the higher incidence (Table III). In 35/48 of the white registries females had the higher incidence, a result which is significant ($P < 0{\cdot}01$). The tendency for non-white males to have the higher incidence was not significant.

The category of "non-white" is clearly unsatisfactory because of the heterogeneous nature of its members; much more useful information can be obtained by the sub-division into the racial groups shown in Tables IV and V. The incidence rates of these non-white populations are frequently based on very small tumour numbers (often less than 5) and are potentially very

TABLE I.—*The incidence of melanoma per* 100,000 *among* 48 *white and* 26 *non-white populations**

| | Mean ± s.e.* | | Student's | |
|---|---|---|---|---|
| | Whites | Non-Whites | $t$ | $P$ |
| Male | 2·634 ± 0·252 | 0·839 ± 0·145 | 4·932 | < 0·001 |
| Female | 3·157 ± 0·289 | 0·761 ± 0·153 | 5·780 | < 0·001 |
| Male plus female | 2·896 ± 0·192 | 0·800 ± 0·104 | 9·774 | < 0·001 |

* The calendar period of registration was not identical for all registries, but was within the range 1960–73, and was most common for 5 successive years.

TABLE II.—*The all-sites* incidence per* 100,000 *among white and non-white populations*

| | Mean ± s.e. | | Student's | |
|---|---|---|---|---|
| | Whites | Non-whites | $t$ | $P$ |
| Male | 221·744 ± 5·728 | 205·016 ± 14·824 | 1·231 | N.S. |
| Female | 193·329 ± 6·317 | 173·879 ± 9·633 | 1·723 | N.S. |
| Male plus female | 207·535 ± 4·466 | 189·448 ± 8·941 | 2·022 | < 0·05 |

* All sites excluding non-melanoma skin tumours.

TABLE III.—*Analysis of the difference in melanoma incidence between the sexes at each registry*

| | Whites | Non-whites |
|---|---|---|
| Registries with "Male" greater | 13 | 16 |
| Registries with "Female" greater | 35 | 10 |
| $\chi^{2*}_{(1)}$ | 9·19 | 0·96 |
| $P$ | < 0·01 | N.S. |

* Yates's correction used.

variable. Restricting analysis to those registries with large tumour numbers, and hence less variable incidence rates, would impose a selection bias. Fig. 3 shows that selecting registries by an increasing minimum tumour number produces a progressively higher mean incidence.

These problems can be circumvented by using the assumption implicit in the calculation of a mean: that the registries of a group can be treated as samples taken from the same population. Thus one can combine the observed numbers of tumours and population sizes, by 5-year groups, to obtain the age-specific incidence rates for the composite population and hence calculate an age-standardized incidence rate. This method is only valid because of the well-defined nature of each registry's base population. One consequence of this method is that the contribution of each registry to the combined rate will be

FIG. 3.—The mean melanoma incidence (per 100,000 population) of groups selected from 48 white populations on the basis of having more than a set minimum number of melanoma tumours (males ——; females – – –).

affected by its population size and by the number of melanomas recorded, *i.e.* the most weight is given to the least potentially variable incidence rate. An indication of the latitude of these groupings of registries was obtained in an analogous way by weighting the latitude of each registry by the total observed population (males and

TABLE IV.—*Melanoma incidence per* 100,000 *of combined registries*

| Registry group | No of registries | Combined standardised* incidence (actual no. tumours) Male | Female | Weighted average† latitude |
|---|---|---|---|---|
| Indians | 2 | 0·18 (19) | 0·18 (12) | 18·1 |
| Chinese | 3 | 0·59 (19) | 0·26 (8) | 5·2 |
| Japanese | 4 | 0·33 (36) | 0·16 (22) | 35·1 |
| All Asians inc. Singapore Malays | 10 | 0·31 (74) | 0·18 (42) | 22·5 |
| Africans in Africa | 2 | 0·96 (13) | 2·29 (22) | 9·1 |
| Africans in U.S. | 3 | 0·68 (12) | 0·61 (14) | 40·5 |
| All Africans inc. Kingston, Jamaica | 6 | 0·95 (38) | 1·03 (49) | 20·8 |

* Age standardized to World Standard Population.

† The latitudes were weighted by the total observed population (males + females × years of observation).

females × years of observation) and taking the average of the weighted values. The Asian and African populations are distributed over a similar range of latitudes, and neither emerges as being markedly closer to the equator (Table IV).

A constant low incidence of melanoma was observed among the sub-groupings of Asian populations, with the highest incidence among the Chinese and the lowest among the Indians (Table IV). In contrast, populations of African descent showed much variation, with the incidence low in North America and high in Africa. However, all groupings of Africans had a higher incidence of melanoma than any group of Asians. No clear predominance of either sex among these racial groups emerges from these data, although the combined rate for all Asian males was nearly twice that of females.

The melanoma incidence rates and the latitudes of the African and Asian populations were also investigated using a non-parametric statistical test. The Wilcoxon rank sum test:

| | No. of registries | Melanoma rank sum M | F | Latitude rank sum |
|---|---|---|---|---|
| Asian registries | 10 | 57 | 58 | 76 |
| African registries | 6 | 79 | 78 | 60 |

reveals that the higher melanoma incidence among Africans is highly significant ($P < 0{\cdot}01$), whereas the distributions of the latitudes of the African and Asian populations are not significantly different.

Details of the distribution of the melanoma tumours among the 4 body regions (head and neck, upper limb, lower limb, and remainder) are available for only some of the registries for non-whites. The category "remainder" is difficult to interpret since it includes those tumours which are "site unspecified", the number of which may vary with efficiency of registration. However analysis of the data for the 3 sites "head", "upper limb" and "lower limb" reveals that virtually all the tumours among Africans occur on the lower limb, whereas among Asians tumours occur frequently on all body sites (Table V).

The incidence of melanoma among other non-white populations (shown in Table VI) is difficult to interpret, either because of the very low tumour numbers involved (*e.g.* New Mexico Indian) or because of the extremely heterogeneous nature of the populations (*e.g.* Cuba and Puerto Rico). It is interesting to note the consistent, relatively high incidence of melanoma recorded by the 3 South American registries in which populations are largely a mixture of Spanish or Portuguese and American Indians. The tumours among these South American populations are found at all body sites, no one site emerging as predominant. The mixed Negro and European populations of Puerto Rico and Cuba show a lower incidence of melanoma than the South Americans, but Puerto Ricans show a similar site distribution of tumours (Table VI).

TABLE V.—*The absolute number of melanomas on the various body sites among Asians and Africans*

| Registry | Male: Lower limb | Male: Head + upper limb | Male: Remainder | Female: Lower limb | Female: Head + upper limb | Female: Remainder |
|---|---|---|---|---|---|---|
| India, Bombay | 5 | 4 | 8 | 5 | 2 | 4 |
| San Francisco, Bay Area, Chinese | 1 | 1 | 0 | 0 | 0 | 0 |
| Japan, Miyagi (prefecture) | 3 | 7 | 2 | 4 | 1 | 1 |
| Japan, Osaka (prefecture) | 3 | 5 | 13 | 1 | 5 | 8 |
| All Asians | 12 | 17 | 23 | 10 | 8 | 13 |
| Rhodesia, Bulawayo African | 3 | 0 | 0 | 3 | 1 | 0 |
| California, Alameda County, Black | 2 | 0 | 0 | 1 | 0 | 1 |
| San Francisco, Bay Area, Black | 3 | 0 | 2 | 4 | 0 | 1 |
| Detroit, Black | 4 | 0 | 1 | 3 | 0 | 4 |
| All Negroes | 12 | 0 | 3 | 11 | 1 | 6 |

TABLE VI.—*Melanoma among other non-white populations*

| Registry | | Incidence per 100,000 | No. of tumours: Lower limb | No. of tumours: Head + upper limb | No. of tumours: Remainder | Total |
|---|---|---|---|---|---|---|
| Brazil, Recife | M | 1·57 | —* | — | — | 18 |
| | F | 1·24 | — | — | — | 16 |
| Brazil, Sao Paulo | M | 2·19 | 13 | 13 | 21 | 47 |
| | F | 1·90 | 18 | 10 | 15 | 43 |
| Colombia, Cali | M | 2·08 | 8 | 5 | 5 | 18 |
| | F | 1·99 | 6 | 9 | 11 | 26 |
| U.S. New Mexico, | M | 0·7 | 0 | 0 | 1 | 1 |
| American Indian | F | 1·2 | 0 | 1 | 0 | 1 |
| Cuba | M | 0·46 | — | — | — | 87 |
| | F | 0·3 | — | — | — | 49 |
| Puerto Rico | M | 0·72 | 19 | 14 | 9 | 42 |
| | F | 0·77 | 25 | 11 | 11 | 47 |
| Israel, non-Jews | M | 0·67 | 0 | 2 | 1 | 3 |
| | F | 0·13 | 0 | 1 | 0 | 1 |
| Hawaii, Filipino | M | 0·29 | — | — | — | 1 |
| | F | 0·00 | — | — | — | 0 |
| Hawaii, Hawaiian | M | 0·92 | — | — | — | 2 |
| | F | 1·02 | — | — | — | 1 |
| New Zealand, Maori | M | 1·50 | 1 | 1 | 1 | 3 |
| | F | 1·54 | 3 | 0 | 0 | 3 |

* Not recorded.

### DISCUSSION

The melanoma incidence rates analysed in this study probably represent the most reliable information available, yet like much survey data they have their limitations. These problems, which include the extent of under-recording of cases and of duplicate registration of the same individuals, as well as the accuracy of site allocation, have already been discussed in detail (Waterhouse *et al.*, 1976). The tendency for "white" registries with a low melanoma incidence to have a low all-sites incidence suggests that the efficiency of registration (of melanoma and all sites) may be sometimes low. This is unlikely to affect the conclusions drawn here.

This study has revealed the wide range of melanoma incidence experienced by both white and non-white populations, at least some of which may be due to variation in efficiency of registration. The mean incidence among white populations was over 3-fold greater than that of non-whites, supporting the suggestion that skin pigmentation protects against melanoma. However, as other authors have reported (MacDonald, 1959; Lewis, 1967), some non-white populations experienced a relatively high incidence, so that skin colour may not be the only factor governing melanoma incidence.

White females in general had a higher melanoma incidence than white males. This has been observed previously (Magnus, 1973; Lee & Yongchaiyudha, 1971) and results principally from the much higher incidence of melanoma on the female leg. It is suggested that this reflects differences in habits of dress rather than a greater susceptibility among females.

Non-whites constitute a very heterogeneous group, but sub-division into more homogeneous groupings produced several groups with few members, so that conclusions must be drawn with care. When similar racial groups were combined it was found that the incidence among African populations was higher than among Asians, although the registries of the 2 groups covered a similar range of latitude. The tumours among Africans were almost exclusively on the lower limb, and it is reasonable to suppose that the majority of these will be on the foot, in view of the many studies which have found this (Oettlé, 1966; Camain *et al.*, 1972; MacDonald, 1959; Lewis, 1967; Fleming, *et al.*, 1975; Davies *et al.*, 1968). This has been observed both among blacks in the United States and those in Africa, although these populations may differ in their shoe-wearing habits. Oettlé (1966) suggested that shoe-wearing was accompanied by a decrease in melanoma incidence, and, in support of this, in the present study revealed a higher incidence among those living in Africa, where shoe-wearing may be less frequent. However, in his study of several tribes in Uganda, Lewis (1967) did not find any correlation between shoe-wearing and melanoma incidence, but suggested that the sites of pigmentation spots corresponded with the distribution of melanoma. Clearly if these spots are more common on the feet of Africans this might explain their greater susceptibility to melanoma at this site. Firm conclusions on this matter must await further observations on other populations (preferably with known shoe-wearing habits).

On those body sites other than the lower limb, the incidence of melanoma among Africans was very low. This result is in agreement with the accepted idea that their dark skin colour protects them from intense sunlight. But a low incidence of melanoma has been reported among albino Bantu in the Transkei (Rose, 1973). It is possible that races normally exposed to severe solar radiation have developed other protective mechanisms. One possibility might be the efficiency of the enzyme systems which repair UV-induced damage to DNA, so that races could differ either in the levels of inducibility or of the fidelity of these repair systems.

Asian populations experienced many tumours on the lower limb, but many also occurred at other sites. Although there have been reports of a high incidence of tumours on the foot (Shanmugaratnam & La'Brooy, 1963; Pringgoutomo & Pringgoutomo, 1963), in contrast to Africans these peoples frequently develop melanoma at other sites (Miyaji, 1963; Tansurat, 1963; Pringgoutomo & Pringgoutomo, 1963; Paymaster *et al.*, 1971). The higher incidence of melanoma among Africans than among Asians in this study was due to the greater frequency of tumours on the foot in Africans; at sites exposed to sunlight the Asians had the higher incidence. Thus a clear correlation between the degree of skin pigmentation and the incidence of melanoma on exposed sites is apparent for the 3 broad categories White, Asian and Negro. This suggests that solar exposure is a major cause of melanoma among Whites and Asians and that melanin pigmentation of the skin is protective. This result raises several further questions: what is the cause of melanoma of the foot; why are Africans so much more susceptible to it; can this unknown factor (or these factors) operate at other sites; to what extent does it or do they operate on Asians and Europeans?

The possibility that at least some of the melanoma among Asians is due to solar exposure is strengthened by 2 reports of a negative correlation between latitude and skin cancer incidence and mortality in Japan (Segi, 1963; Miyaji, 1963). There is clearly much similarity between Asians and Caucasians, since the latter also experience a high frequency of tumours on other body regions than the foot (Magnus, 1973; Lee & Yongchaiyudha, 1971; Davis *et al.*, 1966) and show a negative correlation between incidence and latitude (Lancaster, 1956; Elwood *et al.*, 1974). It is interesting that Caucasians and Asians are also similar in the arrangement of the melanosomes in the keratinocytes, and in the changes which occur in this distribution after UV exposure (Toda *et al.*, 1972). If Asians are susceptible to the solar induction of melanoma, customs of dress may be important, as with Europeans. It is thus possible that if the traditional all-covering dress of many Asian countries is replaced by the more revealing Western styles there may be a rise in melanoma incidence.

These observations suggest that among all brown-skinned races exposed to sunlight one would expect a low incidence of melanoma (compared to white populations at a similar latitude) and that the tumours should occur at all body sites. The somewhat limited evidence from other non-white populations fits these predictions. The consistently high incidence among South American Indians may be due in part to the large percentage of Portuguese and Spanish (*i.e.* white) genes in these populations.

This study has clearly demonstrated that the intensity of skin pigmentation is inversely related to melanoma incidence. This provides strong support for the 2 hypotheses that UV radiation is a major cause of malignant melanoma and that melanin pigmentation protects against this. Additional research is required to elucidate the aetiology of those tumours occurring on the soles of the feet principally among Africans, but also among Asians.

I would like to thank Drs A. Minawa and J. A. H. Waterhouse for helpful advice during the preparation of this manuscript. This work was supported by a grant from the Cancer Research Campaign.

### REFERENCES

Camain, R., Tuyns, A. J., Sarrat, H., Quenum, C. & Faye, I. (1972). Cutaneous Cancer in Dakar. *J. Natl Cancer Inst.*, **48**, 33.

Davies, J. N. P., Tank, R., Meyer, R. & Thurston, P. (1968). Cancer of the integumentary tissues in Ugandan Africans. *J. Natl Cancer Inst.*, **41**, 31.

Davis, N. C., Heron, J. J. & McLeod, G. R. (1966) Malignant melanoma in Queensland. Analysis of 400 skin lesions. *Lancet*, ii, 407.

Elwood, J. M., Lee, J. A. H., Walter, S. D., Mo, T. & Green, A. E. S. (1974) Relationship of melanoma and other skin cancer mortality to latitude and ultraviolet radiation in the United States and Canada. *Int. J. Epidemiol.*, **3**, 325.

Fleming, I. D., Barnawell, J. R., Burlison, P. E. & Rankin, J. S. (1975) Skin Cancer in Black Patients. *Cancer*, **35**, 600.

Gates, R. R. & Zimmerman, A. A. (1953) Comparison of skin with melanin content. *J. Invest. Dermatol.*, **21**, 339.

Gellin, G. A., Kopf, A. W. & Garfinkel, L. (1969) Malignant Melanoma: a controlled study of possible associated factors. *Arch. Dermatol.*, **99**, 43.

Lancaster, H. O. (1956) Some geographical aspects of the mortality from melanoma in Europeans. *Med. J. Aust.*, i, 1082.

Lancaster, H. O. & Nelson, J. (1957) Sunlight as a cause of melanoma. *Med. J. Aust.*, i, 452.

Lane-Brown, M. M., Sharpe, C. A. B., Macmillan, D. S. & McGovern, V. J. (1971) Genetic predisposition to melanoma and other skin cancers in Australia. *Med. J. Aust.*, i, 852.

Lee, J. A. H. & Yongchaiyudha, S. (1971). Incidence of and Mortality from Malignant Melanoma by Site. *J. Natl Can. Inst.*, **47**, 253.

Lewis, M. G. (1967) Malignant melanoma in Uganda, *Br. J. Cancer*, **21**, 483.

MacDonald, E. J. (1959) Malignant Melanoma among Negroes and Latin Americans in Texas. In *Pigment Cell Biology*. Ed. Gordon, M., New York: Academic Press. p. 171.

MacDonald, E. J., McGuffee, V. & White, E. (1973) Status of Epidemiology of Melanoma 1971. In *Pigmentation: Its Genesis and Biological Control*. Eds. V. J. McGovern and P. Russel. Basel: Karger. p. 222.

Magnus, K. (1973) Incidence of Malignant Melanoma of the Skin in Norway, 1955–1970. *Cancer*, **32**, 1275.

Mitchell, R. E. (1963) The effect of prolonged solar radiation on melanoncytes of the human epidermis. *J. Invest. Dermatol.*, **41**, 199.

Miyaji, T. (1963) Skin Cancers in Japan: a nationwide 5 year survey, 1956–1960. *Natl Cancer. Inst. Monogr.*, **10**, 55.

Oettle, A. G. (1966) Epidemiology of Melanoma in South Africa. *Structure and Control of the Melanoncyte*. Eds. Della Porto, G. & Mülbock, O., Berlin: Springer-Verlag. p. 292.

Paymaster, J. C., Talwalkar, G. V. & Gangadharan, P. (1971) Carcinomas and malignant melanomas of the skin in Western India. *J. R. Coll. Surg. Edinb.*, **16**, 166.

PRINGGOUTOMO, S. & PRINGGOUTOMO, S. (1963). Skin Cancer in Indonesia. *Natl Cancer Inst. Monogr.*, **10**, 191.

ROSE, E. F. (1973) Pigment variation in relation to protection and susceptibility to cancer. In *Pigmentation: Its Genesis and Biological Control.* Eds. V. J. McGovern & P. Russel. Basel: Karger. p. 236.

SEGI, M. (1960) *Cancer mortality for selected sites in 24 countries* (1950–1957). Department of Public Health, Tokohu University School of Medicine, Sendai, Japan.

SEGI, M. (1963) World incidence and distribution of skin cancer. *Natl Cancer Inst. Monogr.*, **10**, 245.

SHANMUGARATNAM, K. & LA'BROOY, E. B. (1963) Skin Cancer in Singapore. *Natl Cancer Inst. Monogr.*, **10**, 127.

STARICCO, R. J. & PINKUS, H. (1957) Quantitative and qualitative data on the pigment cells of adult human epidermis. *J. Invest. Dermatol.*, **28**, 33.

SZABO, G. (1967) The Regional Anatomy of the Human Integument. *Phil. Trans. R. Soc.*, **252**, 447.

SZABO, G., GERALD, A. B., PATHAK, M. A. & FITZGERALD, T. B. (1969) Racial differences in the fate of melanosomes in human epidermis. *Nature*, **222**, 1081.

TANSURAT, P. (1963) Regional incidence and pathology of skin cancer in Thailand. *Natl Cancer Inst. Monogr.*, **10**, 71.

TODA, K., PATHAK, M. A., PARRISH, J. A. & FITZPATRICK, T. B. (1972) Alteration of racial differences in melanosome distribution in human epidermis after exposure to UV light. *Nature, New Biol.*, **236**, 143.

WASSERMAN, H. P. (1974) *Ethnic Pigmentation—Historical, physiological and clinical aspects.* Ch. XI, p 119. Amsterdam: Excerpta Medica.

WATERHOUSE, J. MUIR, C., CORREA, P., POWELL, J. & DAVIS, W. (1976) *Cancer Incidence in Five Continents*, Vol. III. Lyon: IARC Scient. Publ. **15**, Lyon: IARC.